# “They connected me to my health”: perceived benefits of an etiological cohort study led by community health workers in the Southwest Borderlands

**DOI:** 10.3389/fpubh.2026.1736316

**Published:** 2026-05-11

**Authors:** R. M. Crocker, K. R. Duenas, M. Ingram, M. Figueroa, E. Torres, S. C. Carvajal

**Affiliations:** 1 Department of Health Promotion Sciences, University of Arizona, Tucson, AZ, United States; 2 Campesinos Sin Fronteras, Somerton, AZ, United States

**Keywords:** community health worker (CHW), immigrant health, medical care access, relational healing, stress, therapeutic landscape theory, US-Mexico border, study experience

## Abstract

**Introduction:**

Community health workers occupy an increasingly prominent role in the broad sweep of community engaged and participatory action research approaches that center equitable and inclusive research practices and prioritize community needs. This study utilizes the theoretical lens of therapeutic landscape to explore the ways in which community health worker-led research may impact study participants based on data from a closing interview conducted among Mexican origin participants engaged in an etiological cohort study of stress and resilience along the US-Mexico border.

**Methods:**

This study builds upon a longstanding partnership between the University of Arizona and a community farmworker service organization to focus on chronic disease and mental health in Yuma County, Arizona. The community health workers were involved in all phases of this mixed methods study, including conception, design, methodology, data gathering, analysis, and dissemination. We recruited 282 Latino/a adult residents of southern Yuma County, Arizona using a randomized household door to door approach and data gathering occurred between March 2022–April 2024. The open interview question that informed this article was conducted among a subset of those who participated in the final timepoint of data collection (*n* = 192) thematically analyzed by study team members and descriptive statistics were calculated in R Statistical Software, Version 4.2.0 “Vigorous Calisthenics” for participant demographic characteristics.

**Results:**

Our findings suggest that participants experienced multiple benefits derived from their research interactions with CHW-Rs. Notably, these gains spanned several areas ranging from the immaterial experiences of emotional support and opportunities for novel self-reflection to the practical acquisition of health information and individualized health results. Taken in concert, participants reported that positive study experiences mobilized them to take tangible action to improve their own wellbeing in the areas of improved dietary and physical activity practices, greater rates of access of medical care, and prioritization of mental health needs.

**Discussion:**

Our analysis identified an important relational component of participants’ study experience that centered around how the community health workers developed an environment of care and trust with participants that inspired deep sharing and reflection and receptivity of new health information, all of which mobilized them to improve their health. These results imply the potential for participants to derive substantial gains in the context of both interventional and observational research which can help to bolster the inclusion of immigrant and displaced populations in the research endeavor.

## 1 Introduction

Community health workers occupy an increasingly prominent role in the broad sweep of community engaged and participatory action research approaches that center equitable and inclusive research practices that prioritize community needs and education (1, 2). Community health workers (CHWs) are defined internationally as “frontline public health workers” who are members of the communities where they serve and share a common language and ethnicity (3, 4). Their inclusion in research has been heralded for improving the prevention and management of chronic disease and effectively communicating health information, and in 2016 research and evaluation were added to CHWs core competencies (5–7). Moreover, CHWs who participate as researchers, known in the field as CHW-researchers (CHW-Rs), contribute to the translation and long-term sustainability of research in community settings, via their ability to incorporate the specific health knowledge and leadership skills gained into their ongoing work, organizations, and local ecologies (8).

From the vantage point of the research enterprise, CHW-Rs provide a significant value-added component to a broad range of interventional and observational research studies. CHW-Rs use their extensive social networks and ability to build rapport to increase participant recruitment, retention, engagement, and follow-up, as well as to inform research design (9, 10). They also leverage their cultural knowledge to tailor and improve the quality of data collection instruments thus contributing to their cultural and contextual relevance (11, 12). In community centered studies, CHW-Rs often serve as community representatives with academic partners and work to build mutual understanding and respect between researchers and community members and to ensure community perspectives are prioritized (8, 13).

However, CHW-Rs have also been shown to offer other direct though often less material benefits to individual study participants. In their profession, CHW-Rs address community members’ lives holistically, and thus are inclined to identify participants’ non-clinical needs and offer them support in accessing community resources (14, 15). In this way, CHW-Rs have been found more broadly to “enrich the quality of life for people in poor, underserved, and diverse communities in the United States” (16, p. 179).

While these factors benefit all research participants, they may be of particular importance to those from immigrant and non-English speaking backgrounds who face added barriers to integration into vital health and service systems due to immigration status, language and other factors (17, 18). CHW-R’s ability to effectively communicate with participants based on their local knowledge, communication skills, and bilingualism is a driving force in their inclusion in research (1). In addition, “promotoras de salud,” as they are known in Spanish, have a rich history of deep community engagement along the US-Mexico border and in many Latin American countries, where they often fill important gaps in medical care systems (19, 20).

In this article we apply Gesler’s (21) theory of “therapeutic landscape” as a means to expand our understanding of how and why studies that partner with CHWs as researchers may offer increased benefits to research participants from immigrant backgrounds. Therapeutic landscapes are broadly understood as geographically, socially, and symbolically defined “spaces” that facilitate healing (21). While originating in social geography to study the unique physical attributes of healing destinations, the concept of therapeutic landscape has since spread across the humanities and social sciences because it also centers the social and symbolic components of spaces that promote recovery and restoration (22, 23).

In foregrounding the healing potential of environments, therapeutic landscapes are understood to operate via the lens of human perception and emotional ties in the context of multifactorial lived experience (21, 24). Current scholarship addresses “the geographies of health maintenance in everyday life” (25), p. 122, such that any place (including a private residence or medical care setting) that is at once bound by space but transformed by human interaction can be subjectively perceived of as a healing landscape (26–28, 73).

Here, we operationalize therapeutic landscape theory to highlight an exploratory analysis of perceived benefits associated with participation in a CHW-led research study among Mexican origin participants engaged in a study of sources of stress and resilience along the US-Mexico border. While these data are based on brief responses that do not capture comprehensive participant experiences, they bolster an argument for additional study of the full range of CHW impacts on study interactions, even in the absence of interventional study components. As CHWs become increasingly centered in the research enterprise, these data will be critical for their training and development as community engaged researchers as well as for researchers’ ability to holistically examine study impact.

## 2 Materials and methods

### 2.1 Study design and community context

The longitudinal study was developed in the context of a longstanding partnership between the University of Arizona Center for Participatory Evaluation and Action Research and the community organization Campesinos Sin Fronteras with a focus on chronic disease and mental health in Yuma County, Arizona. Campesinos Sin Fronteras is a farmworker health, rights, and advocacy organization with deep history of direct service provision and community centered research (13). The focus on stress and resilience builds upon on-going community interest to address mental and behavioral health issues that exist among the US-Mexican border towns of San Luis and Somerton, Arizona. Central to the partnership is mutual trust in considering the burden and benefits of research for community members and respecting the needs of the community in the research process. The current article is based on analysis of one question that was included as part of a closing interview implemented during the third time point of this longitudinal study in response to the community organization’s interest in an evaluation of the study’s community impact.

A key characteristic of our CBPR approach involves ensuring that Campesinos Sin Fronteras CHW staff were central to the research decision making process and active across the research enterprise, which has been identified as crucial for ethical and meaningful engagement in immigrant communities (18). Beginning with method’s development, Campesinos Sin Fronteras’s vast experience in community engagement provided essential insight into appropriate recruitment and participant retention approaches as well as in defining key study terms like stress and resilience in the community context. Campesinos Sin Fronteras’s contributions to this process stems from decades of grassroots experience working within the southern Yuma County community and set the stage for the smooth and successful implementation of the research methodology.

Methodology for the longitudinal study included both qualitative and quantitative phases designed first to explore and then measure factors contributing to resilience, stress and participant relationships to stress and chronic disease [for full study protocol, see (29)]. The research team integrated the complementary knowledge and expertise of the University of Arizona and community partner researchers by conducting co-analysis activities grounded in our shared reflexivity to inform data interpretations and dissemination. The team’s commitment to enacting principles of “relational publishing”—“building trust, capacity, partnership, and knowledge together while retaining authorship of storytellers and artists themselves” (30), p. 213—have resulted in four co-authored articles and a dedicated and fully bilingual journal issue in which several CHW-Rs led their own narratives reflecting on the study experience [see (11, 13, 31–35)]. The CHW-R’s testimonies of their own personal transformations and growth during the study further attest to the relational nature of the study experience for all parties.

### 2.2 Data gathering

In the second and longitudinal phase of the study, we recruited 282 Latino/a adult residents of Somerton and San Luis using a randomized household door to door approach. The CHW-Rs engaged with research participants over the course of one year to examine stress, resilience, and psychosocial protective mechanisms through 45-min self-report surveys, biospecimen collection to assess objective biomarkers of stress, and a brief closing interview added to the end of timepoint 3.

The CHW-Rs recruited participants from March 2022–March 2023 and performed data collection on a rolling basis dependent on the timelines of each individual participant. Data gathering occurred between March 2022 and April 2024 during which the COVID-19 pandemic significantly impacted US-Mexico border communities. The CHW-Rs administered the surveys in the participants’ native language (Spanish) across three time points, each 6 months apart. The participant retention rate over the 12- month study was 70.6% (time point 1, *n* = 282, time point 2, *n* = 245, time point 3, *n* = 199). This attrition rate is considered good for a CBPR study, particularly when carried out among challenging data gathering conditions, such as with immigrant and farmworker populations that are more mobile than others and also considering the challenging context of the COVID-19 pandemic (36, 37).

For the first interview at time 1 (baseline), the CHW-Rs visited the participants’ home setting, while for the six and 12 month follow ups participants traveled to Campesinos Sin Fronteras to conduct the appointments in a private office room. Participants received point of care results at each time point, including cholesterol level, glucose, and blood pressure and CHW-Rs provided basic health education in response to participant questions.

The trust and respect that the CHW-Rs have in the community helped build significant rapport among participants over the course of research, such that participants’ responses oftentimes expanded beyond the predetermined multiple-choice options. When a participant demonstrated a need for additional support or services, the CHW-Rs provided relevant referrals to local health and service providers.

#### 2.2.1 Ethical statement

This research study was approved by the Institutional Review Board of the University of Arizona (# 2003462440).

### 2.3 Closing interview

The closing interview was added to the third time-point at the request of the CHW-Rs who had heard many personal stories from participants about how the study and their interactions with the CHW-Rs had benefitted them. The CHW-Rs wanted to evaluate the perceived experiences and impact of study involvement on participant’s health related behaviors, as well as inform future programming that would be responsive to study findings about stress and resilience in the community. A key component of CBPR methodology is being responsive to the needs and interests of the community organization, and the closing interview offered Campesinos Sin Fronteras the opportunity to further their own growth as a research-driven organization by enhancing the CHW-Rs’ research skills and initiative in shaping future CBPR studies.

The first author developed seven questions for the closing interview using notes from weekly team discussions which captured the CHW-Rs reflections on participant interactions. The closed and open-ended questions were edited and translated to Spanish for cultural and regional appropriateness by the research team. The CHW-Rs conducted the closing interview in the style of an open-ended interview after participants completed the third survey. Because the closing interview was added just after initiation of timepoint 3, it was conducted among 192 of the total 199 participants who responded to timepoint 3. The CHW-Rs documented word for word responses in RedCap mobile on iPads. This article incorporates data fom the open-ended question “Please tell us a little bit about your experience being part of this research study,” and answeres were generally between one and three sentences long.

The decision to have the same CHW-Rs who interacted with participants throughout the study conduct the closing interview was an intentional strategy to build on their sustained engagement with participants that had generated substantial rapport and trust in discussing sensitive data. However, this data collection strategy had the potential to introduce “social desirability bias,” a common issue in qualitative and survey driven data in which participants seek to answer in a way that sounds positive and matches societal expectations, particularly around sensitive issues (38). Our team made several important efforts to reduce the potential for social desirability bias to influence participants’ responses.

First, building on established rapport has been shown to reduce social desirability bias (39), and the CHW-Rs in this study had already observed that over the course of the study, participants were increasingly open about their personal experiences related to health and other challenges. In addition, the CHW-Rs reiterated the importance of providing honest answers to study questions across all phases of the study. Prior research has similarly found CHW’s ability to establish an environment defined by emotional safety, culturally-rooted respectful communication, and linguistically consonant care works to cultivate trust and reduces power imbalances between community members and health providers (40).

In addition, we ensured that participants had opportunities to provide anonymous feedback over the course of the study by providing the principal investigator’s direct contact information on the written informed consent, a copy of which was left with all participants at their initial research appointment. Several members of university research team who were not actively involved in data gathering also traveled to the study site on multiple occasions to engage with participants and reinforce that they could reach out to us directly with any issues or concerns. We did not receive any responses via either of these mechanisms. Lastly, an important means to reduce the potential for social desirability bias to influence survey or interview findings is by triangulating with observational data (41). The CHW-R’s had observed that participants were commonly reporting back informally about the study benefits throughout data gathering visits, which served as the initial impetus for carrying out the closing interview and offered an important means to ground closing interview findings (34, 42).

### 2.4 Analysis

In Fall 2024, a rapid qualitative analysis of the closing interview responses was conducted to share initial findings with the research team before the study’s conclusion. We selected this method for its practical application as a means to share actionable and informative qualitative findings on shorter timelines that benefits communities and community-based organizations seeking to develop programming and outreach that are response to research results (43, 44). Moreover, the rapid qualitative analysis approach emphasizes team reflection and reflexivity to interpret data which is appropriate in cases where the data may not consist of long interviews but is built on extended rapport among participants and research teams and is also suited to inform actionable steps for community organizations that work to address rapidly evolving and stressful environmental conditions, such as the COVID-19 pandemic (45, 46).

To conduct the rapid qualitative analysis, the third author first reviewed all the responses by question and categorized them by general themes in a Word document, which is a documented approach to qualitative group analysis of short open-ended text in a survey (47, 48). She then read through each theme and created sub themes that further defined the responses, coding all the data under the subthemes until theme saturation was reached. The second author then conducted cross-coding by reviewing the data and the themes and recommended minor revisions to the codes. The coders then conducted member checking by presenting the analysis findings back to the whole research team to ensure that the codes identified matched with the themes identified by the CHW-Rs during data gathering and as well as by their informal observations and conversations with study participants over the course of the data gathering process. This iterative process served as a means of triangulation among members of the research team, and no clear disagreements in the coding emerged, indicating the straight-forward nature of the data.

In Summer 2025, the first author reviewed the analyzed data specifically for the question “Tell us a little bit about your experience being part of this research study” and performed a slight re-organization of the initial analysis themes according to the theory of therapeutic landscape. This was a deductive process in which the elements of therapeutic landscape theory—specifically social landscapes—were used to tag thematic groupings in the data related to the perceived healing impacts of the study’s social landscape. In this manner, she re-grouped the initial thematic codes into theoretically informed categories according to the therapeutic landscape lens. She then reviewed this theoretical breakdown with Campesinos Sin Fronteras staff in August 2025 and noted their additional thoughts and responses to the data to ensure cultural relevance and accuracy of data interpretations. The different themes resulting from the phased analysis process are displayed in Table 1. The first author, who is a trained translator and interpreter, translated all quotes in this article and they were then verified by a native speaker from the study team.

**Table 1:** Theme generation during phased analysis of closing interview question.

Phase 1: Rapid qualitative analysis themes	Phase 2: Therapeutic landscape analysis themes
Theme 1: Increased awareness of health and health improvements Exams helped become more conscious of my health I can see improved results Helped me notice what I eat, take care of health, seek information Questions helped me reflect on physical and emotional health Theme 2: Awareness of mental health/importance of reaching out I have sought professional assistance I have learned better stress manage and communicate more I am more aware of the importance of stress Theme 3: The promotoras provided an opportunity to share, provided support and connected them with services I benefited/emotionally They helped link to services Gave opportunity to share I felt heard/supported Theme 4: The focus of the study has been a good/Interesting experience Stress is important to understand on the border Felt good being part of the study, learning and helping others Theme 5: Learning about/responding to emotional health The questions led to personal reflection and growth	Theme 1: Relational—study participation made me feel good, feel cared for, learn to express myself Theme 2: Health information—study informed me about my own health and healthy behaviors Increased awareness of mental health and importance of seeking help Theme 3: Health literacy—learn from *promotoras* about what services are available Theme 4: Encourage self-reflection about physical and emotional health Theme 5: Giving back to community The stress focus of the study is important on border feels good to participate in something positive for all Theme 6: Motivational—study made me take better care of health and seek information Go more to doctors Eat better & exercise more Improve emotional/mental health Decrease stress levels

Descriptive statistics were calculated in R Statistical Software, Version 4.2.0 “Vigorous Calisthenics” (29) for participant demographic characteristics. For the purposes of the current study, we analyzed baseline data for demographic characteristics of participants who completed the closing interview (*N* = 198) such as ethnicity, gender, age, level of education and health insurance status, city of residence, chronic disease diagnosis, employment type, and birth country.

## 3 Study results

The sub-sample of study participants who completed the closing interview included 198 Latino adults living in southern Yuma County, Arizona 77% of whom were female (Table 2). The mean age was 55 years of age at the time the closing interview was conducted, and the vast majority were born in Mexico (93%) and now lived in San Luis, Arizona (96%). Over half the sample (60%) had less than a high school education and the majority (72%) were married. The most common areas of employment included agriculture and caregiving roles. In terms of health, 92% of participants had health insurance and in general they reported a heavy burden of chronic disease, with the most common ailments (in descending order) being cholesterol, hypertension, diabetes, anxiety, gastrointestinal diseases, chronic pain, and depression.

**Table 2:** Sample demographics.

	Time 3	
	(Exit survey *N* = 198)	
	*n*/mean (range)	(%)/SD
Gender		
Female	152	77%
Male	46	23%
Age	55.02 (19.63–84.58)	14.2
Birth country		
US	13	7%
Mexico	185	93%
Education		
Less than high school	119	60%
High school or more	79	40%
Health insurance status		
Insured	171	92%
Uninsured	30	16%
Marital status		
Single	21	11%
Married	142	72%
Divorced	12	6%
Widow	16	8%
Separated	5	3%
Free union	4	2%
Employment type		
Agriculture & field work	57	29%
Caregiving & domestic work	17	9%
Customer facing roles	11	6%
Skilled/Technical work	7	4%
Not employed	106	54%
Chronic disease		
Cholesterol	101	51%
Hypertension	81	41%
Diabetes	69	35%
Anxiety	54	27%
Gastrointestinal diseases	50	25%
Chronic pain	44	22%
Depression	42	21%

This table summarizes demographic characteristics at time point one and three for the study. Demographic variables include gender, age, birth country, city of residence, education level, and health insurance status, marital status, employment type, and chronic disease diagnosis with corresponding means, percentages, and standard deviations (SD) provided.

When asked to reflect on their experiences as being part of the study, participants provided brief responses that broadly indicated positive perceptions about the study. While no overtly negative responses were recorded, three participants did not provide a response, and a few participants provided neutral responses or were unable to articulate their opinions beyond having generally enjoyed being part of the study. For example, one participant simply stated,” It was a good thing; I do not know how to explain it.”

More commonly, however, they reported specific aspects that were beneficial to their lives. Taken in concert, participants’ responses characterized the study experience as having provided a therapeutic social landscape that gave rise to healing and promoted personal agency in health and care seeking. The three primary therapeutic social elements that participants mentioned experiencing during their study visits were: (1) the emotional connections they made with the CHW-Rs who visited their homes, (2) the opportunities these visits and the survey questions gave them to reflect upon their lives and their health, (3) the new knowledge they gained during the study about their own health status and healthy practices more generally. These healing elements appear to have encouraged and motivated many participants to make tangible and changes in their lives that were intended to improve their health, including seeking professional care, improving dietary and physical activity practices, and prioritizing their mental health.

### 3.1 Relational healing as therapeutic social landscape

Participants commonly described having forged strong emotional connections with the CHW-Rs during their study visits, which promoted participants’ sense of trust and made them feel cared for. One participant explained, “I have felt very comfortable, and they come to your home. I have felt good,” while another commented “I feel comfortable sharing time together with you all.” Many repeated that the CHW-Rs treated them very well and with respect and that they always followed through on what they said they would do.

One element of this relational therapeutic landscape was feeling cared for, a social dynamic that took place regardless of whether the study visit occurred in participants’ homes or in the offices of Campesinos Sin Fronteras. Participants expressed appreciation for study staff and the study overall for making them feel that their lives and their health were important to others. One participant explained: “we matter to people, and they help you with the studies that they are doing with us.” Another said: “I like [the study] a lot because they care about my health and my stress. Thank you for this program.”

An important element of this care involved participants feeling supported in taking care of themselves and their families. This ranged from providing a listening ear, encouraging positive changes, giving reminders for study appointments, welcoming them to services at Campesinos Sin Fronteras, and making referrals for additional services. A participant noted: “[The study] changed my life because they paid attention to me and the changes I am making in my life. And the tests they are doing remind me that I need to be getting those tests done periodically.”

A critical aspect of the therapeutic social landscape described by participants was the social support offered by the CHW-Rs. One participant explained: “[The CHW-Rs] came to my house and they have supported me a great deal in my way of thinking and in feeling that I now have someone to whom I can look for help if I need it.”

The fact that the study took place amidst the isolation and loneliness induced by the COVID-19 pandemic accentuated participants’ needs for social connection and elevated the healing impacts these personal connections had in their lives. Participants expressed appreciation for the fact that the CHW-Rs carried on with the study during a time when many activities and service provision were halted. One said: “It is really lovely because there still are good people that are willing to help others.” Another participant expressed: “I really liked being able to talk with someone since my family closed ourselves in during the pandemic and we did not have contact with anyone.”

#### 3.1.1 Sharing their problems provided relief and relaxation

Participants also cited that the caring social environment established by the CHW-Rs was therapeutic because it helped them feel safe expressing themselves and talking about their problems. For some who had lacked prior opportunities for sharing their feelings, this was a novel experience. One participant noted, “Yes. I liked [the study] because I learned how to express myself and I saw how I was doing with my health.” Another echoed: “I feel quite fortunate to have the experience of living what it is like to have communication with others. It has also helped me to progress a bit about my stress.”

Another participant spoke at length about how the study had reinforced the importance of sharing, noting that Latino cultural norms could encourage people not to share their troubles, saying: “I have opened up a lot because I’m a person who tried to keep it all inside and who is now trying to talk more. I have told people about this program, but many people from our culture, I think it makes us keep things inside and not want to express ourselves and I have told them that [talking] helped me a lot because I opened my mind.”

A common phrase used by participants to describe the study impact was “te desahogas,” meaning “you get things off your chest.” One participant said participating in the study was “Really great [with] excellent care and you get the opportunity to share your problems and be heard.” Another mentioned that “talking makes one feel like you can express yourself or you unburden yourself.” The trust established with the CHW-Rs was instrumental to this sharing, and one participant remarked that “talking [to the CHW-Rs] is as if I was talking with a friend to unburden myself.”

Many participants commented that the results of being able to share this burden with others was that they felt noticeably calmer, more relaxed, and supported. One said: “It has really helped me a lot, I come away relaxed and happy.” Another participant explained: “It has helped me a lot, sometimes we need to talk about things to feel better; sometimes when I have problems, I just remember the survey.”

### 3.2 Opportunity for personal reflection as therapeutic social landscape

Another key element of how the study offered a therapeutic social landscape was affording participants the time and motivation to reflect on aspects of their lives that they had not fully considered before. This reflection was the result of being asked the survey questions across the course of a year and considering how their answers may have shifted over time. Participants mentioned that their reflections had a particularly strong impact on how they viewed their own mental and emotional health and as well as that of their larger communities.

#### 3.2.1 Reflection led to personal growth and understanding themselves

Many participants perceived that the opportunity to reflect on the survey questions encouraged them to examine underexplored aspects of their lives in a way that promoted personal healing and growth. One participant noted: “Well I like that they ask me [questions] because that is how I learn how I am doing emotionally and physically and that reflects what [issues] I have and who I am.” Another explained: “I think [this study] can help people because we do not really think about these questions until they asked them to us.”

Some said that the reflections helped them focus on specific issues or problems they had. Others noted that the experience of being repeatedly asked the questions opened their mind to consider how they were doing more generally and no longer deny problems that they had previously tried to avoid. One participant explained: “when I got home, I thought a lot about how I had answered the questions because there are times when one says that they do not have any problem when they actually do so there are times when one needs help, but they deny it or say they do not need it when they do. And well, in this study I learned a little bit more about myself.”

Participants identified these reflections as the basis for subsequent changes they described making in their lives (see section 3.4). For some, these changes necessitated seeking professional care or participating in programs in their community, while others described ways that they instituted changes in their daily lives. One participant said: “This study seems really good to me because I learned things that I would not have known otherwise, and I started to work [on my problems] and I think this has produced results.”

Another participant described how the study made her reflect on how much she had been prioritizing work to the detriment of her family life. She said: “It encouraged me to be a bit closer with my family, because at first, I always was, like, more focused on work. I started to reflect on this and changing jobs so I could spend more time with my family.”

#### 3.2.2 Reflection increased awareness of mental health needs

Many participants perceived that the opportunity the study offered for reflection had an especially notable impact on their mental and emotional health. Some participants said that the study highlighted their mental health needs for the first time, often helping them to identify unrecognized needs. Others reflected on specific stressors in their lives that had been compounded by the loss of loved ones and work and fear of contagion during the COVID-19 pandemic.

One participant said: “participating in the study really excited me because it will help people who need help, who feel stressed and depressed; and [it made] me realize that I get stressed, which I did not know.” Another identified mental health conditions during the study that had a profound impact on her life: “Thanks to the survey questions, I was encouraged to get better, since I realized that I had stress, depression, and anxiety, and I sought out psychological help. When they left the [study recruitment] paper on my front door, I felt that it was a call from the universe since I was feeling depressed.”

Participants mentioned reflecting more on their relationships with other people, especially family members and partners, and whether they had sufficient support in their lives. One participant thanked the study for enabling them to examine the way in which they “get along with others, the type of communication I have with people around me and also to know whom to go to for help.”

The focus on stress in the study provided participants with the opportunity to reflect upon the role stress played in their overall health and well-being, as well as in the community at large. A participant explained that “I have realized how important stress is in people’s daily lives.” Another participant focused on the ways stress has impacted border residents specifically, saying: “people need to realize that stress does exist and about how important it is to overcome it and understand that violence has had a big impact in Mexico since the stress [there] is much greater.”

Participants also commonly reflected on their relationship to depression. Several explained how the study had helped them to stop ignoring or covering up feelings of depression and identifying other emotional stressors. One participant said:

The study has helped me because after the interviews I have been able to meditate on each question they asked me about stress and depression, which sometimes one thinks they have not experienced. But really, when they ask you those questions, afterwards you think “yes, I do think I have experienced depression. Maybe not so intensely, not so severely, but yes, some days the truth is I have felt like that.”

#### 3.2.3 Reflections mobilized participants to invest in their community

In the process of speaking with the CHW-Rs about the purpose of the study and how results would be used, many participants reflected on health, stress, and other issues facing their communities and the US-Mexico border region more broadly. Participants described taking a significant interest in expanding their understanding of their community’s health. One participant noted: “I have had a positive experience; many questions investigated the truth about people living along the border and I would like to be able to read about it. It seems very interesting to me.” Another commented: “This [study] is something that helps us to understand our culture better by thinking about the questions that they ask us.”

Many participants expressed feeling pride and satisfaction from being a part of something that they believed would ultimately benefit their communities. One participant described: “I feel good participating in this study. I feel that, in some way, I will help with something.” The feeling of participating in something larger than themselves was gratifying for many participants and encouraged some of them to reach out to friends and neighbors. One participant explained: “I have shared [about the study] with my neighbors, and it has been really good for me. It motivates me a lot and knowing that I am helping through this study makes me feel proud.”

Some participants specifically mentioned that participating in the study inspired them to want to do more to help their community, including participating in future studies of local benefit. One participant stated: “I like that they included me. I like to help people and that my opinion matters. It motivated me to get involved in other studies and programs with Campesinos Sin Fronteras.”

### 3.3 Health and care seeking information as therapeutic social landscape

Study participants also experienced perceived benefits from gaining important information from the CHW-Rs about their own health status as well as about healthy practices care seeking.

#### 3.3.1 Point of care results informed participants about their own health

Most participants mentioned that the opportunity to receive point of care results at each of the three study time points was highly beneficial and greatly expanded their previous understanding of their own health. They described the specific results they received (cholesterol, glucose, and blood pressure), and noted that taken in concert, these results added significantly to their knowledge about their overall physical condition.

One participant explained: “My experience was about realizing what health condition I am in,” while another said: “It has helped me because they check my cholesterol and my triglycerides, and I got a sense of how I am doing.”

Participants frequently expressed gratitude for the study, explaining that getting access to these types of test results without the study would have been expensive or overly time consuming. One gave thanks for the study and offered: “I hope they continue to have funds so they can help more people. I would like that.”

Others mentioned that they had not previously prioritized regular health check-ups and had thus lacked critical information about their own health risks. One participant explained: “I realized that I had about a year since I had done a physical [exam]. I realized that I was pre-diabetic and have high cholesterol.” Another described how the study had fundamentally changed their relationship to their health, saying: “Well I like that they have checked my blood, they have connected me to my health by paying attention to my health during this time and it has gone really well. Thank you for that.”

#### 3.3.2 Gaining health information and healthcare literacy

Participants also expressed appreciation for the information the CHW-Rs shared with them about healthy practices and the importance of seeking health and other services. The study encouraged participants to put their health higher on their radar and list of priorities, as one participant said: “Well it has helped me to take my health into account and pay more attention to what can affect me.”

Participants mentioned learning about the value of seeking regular medical care, reaching out when they need help, best nutritional practices, and tips for staying active and maintaining a healthy lifestyle. One participant explained: “Well one learns about a lot of better ways that really help them move forward. At least for me, I really liked it, *mija*.” Another said: “I can see now that I was wasting time because I did not do any exercise, I did not walk, I ate worse. And now I eat a bit better.”

Others described learning from the CHW-Rs about the importance of seeking regular professional care as well as participating in other family and community activities that could support them. These included services available at Campesinos Sin Fronteras, such as parenting classes and support groups, as well as community activities such as religious services. A participant stated: “this [study] was a really good experience. It helped me to see that I need help and to find it.”

One participant expressed: “Through this program, it has helped me to realize that we have resources and supports in the community for our emotional needs, or social needs, or health needs. And it has also made me realize that we can count on the support of family and friends.” This orientation toward seeking support and care was a fundamental shift for many participants who had previously tried to shoulder all their challenges on their own. Another participant explained: “There is no problem that I alone can manage. And seeing that I need help or that they advise you since they will be more likely to resolve the problems that we have.”

### 3.4 Therapeutic social landscape encouraged healthy action

When asked how the study impacted them, many participants described making tangible changes to improve their health as a results of the therapeutic social landscape they had experienced with the CHW-Rs. They often connected these changes to the experiences described in the preceding sub-sections - to the care they received from the CHW-Rs, the reflections they made on their own lives and well-being, or to the information they learned about their own health and healthy practices. One participant lamented the study’s conclusion, saying: “Being here with you has made me take care of myself more, and truthfully I do not want to leave [the study] with you all. And I was paying such close attention to my health.”

One participant said the study was: “a new experience of trying to improve my life right now into a different situation from who I was before.” For another, the study was a wake-up call: “I felt happy and grateful for what the doctor did not find given that I was not feeling well and I had poor nutrition. And this study has helped make me take better care of my health.”

For many, these changes had resulted in noticeable improvements to their health. As one participant described, “while one is in the study, you take better care of your health because you did not want to come out badly on your cholesterol, sugars, and blood pressure. And I have really felt better.” Another participant noted that: “I have felt good, and it increases my awareness of mental and physical health, and nutrition. Leaving here, I take better care of myself.”

These improvements motivated participants to stay in the study and keep working at their health. As one participant explained: “Well it has helped me a lot. I have felt good, and that’s exactly why I am coming [to the study appointments], because I have seen that I have gotten results and that you all keep helping me with that.” Another participant noted that: “It’s always good to be a part of a study, because it helps you change. It has helped me to take care of my emotional health and to avoid physical and mental illnesses.”

While participants described changes in many areas of their lives, they focused primarily on seeking medical care more frequently and for more diverse purposes, improving practices related to diet and physical activity, and working to address stress, depression, and other mental health issues.

#### 3.4.1 Seeking more frequent medical care

Many participants stated that, thanks to the study, they had started seeking medical care more regularly. One participant explained: “[The study] really changed my life a lot because they paid attention to me, and the changes that I have made in my life with the exams reminds me that I need to be getting tested periodically.” Another simply stated: [The study] makes me think about my health and make changes to my health, going to the doctor more and taking better care of myself.”

While some participants stated having sought medical care for diagnostic purposes, others were made aware during the study that they had chronic and other health ailments that they needed to seek routine care to manage. One participant explained: “This study helped me realize that I had problems with cholesterol, [blood] sugar, and diabetes and I have been going more frequently to the doctor and improving. [I have] been doing exercises and relaxation activities.”

#### 3.4.2 Improving dietary habits and increasing physical activity

Participants frequently stated having worked to improve their nutritional habits and increase their levels of physical activity according to the health information they had received during the study. Many saw these changes as necessary to prevent the onset of illness or improve their current health status. One participant explained: “I really liked this research experiment; it helped me a lot and it made me realize that I need to take better care of how I eat and my weight so that my blood pressure is okay and I come out alright with my triglycerides and my cholesterol.”

Participants mentioned trying to consume less sugar and sodas and increasing their intake of vegetables and other foods considered “natural,” although some lamented that these foods were more expensive to purchase. Healthy eating was described as a critical part of a healthy lifestyle and was often noted in conjunction with exercising more frequently and working to reduce stress. One participant noted: “I have made changes to my diet to prevent diabetes, and I started to walk and in terms of stress I am trying to live a calmer life because it’s important for my health.”

Several participants also mentioned that they had shared the nutritional and other health lessons they learned during the study with other family members. One participant said: “My experience helped me help my children so that they could change their lifestyles in terms of their diet and their way of being as individuals.”

#### 3.4.3 Addressing mental health issues

Lastly, participants described the ways in which study participation had alerted them to the importance of addressing their mental and emotional health needs. They described seeking to address these needs in informal ways and family settings, as well as via seeking professional help. One participant stated: “[The study] has helped me open my eyes about mental health problems and has put me in a position to face them and seek help.”

Some participants responded to their increased awareness of mental health by trying to maintain a more positive outlook and mood, improving their communication with others, and maintaining greater calm in the face of their problems. One participant explained: “I have shifted my way of thinking to a positive one and am not taking my problems so to heart.” Another participant stated: “[The study] has helped me to move through my stress and stay calmer so as to move forward in my life.” One participant described study participation as a time when she “studied herself,” which encouraged her to reach out more to her extended family and improve her family communication style with her children and husband.

Others described having sought professional mental healthcare, often for the first time, in response to their reflections on their own well-being and learning more about help seeking from the CHW-Rs. One participant said: “I have felt good. I had already gone to the clinic, but they had never sent me to a psychologist and here they referred me there, and that helped me a lot.” Another woman described how seeking mental health support had helped her navigate a family crisis, saying: “[This study] is a very good experience. It helped me get beyond the difficult period that I went through with my husband. They helped me to seek help and pull myself together. I thank them for what they have done for me.”

Participants mentioned that seeking care for their emotional and mental health needs had made them feel less alone. One participant recalled: “I did not leave my house. Now I do go out a bit, and I am more sociable with others. I sit at the park and watch. I was able to seek help for my stress, and it helped me a lot.” Others referenced that Latino culture sometimes encouraged people to bury their problems and resist being vulnerable and allowing others to see your pain. A participant described previously overlooking symptoms of depression, but that “with the classes I have taken with you and these interviews, I have realized that it is necessary to talk and to know what one is feeling.”

## 4 Discussion

Our exploratory analysis of perceived benefits associated with participation in a CBPR research study among Mexican origin participants along the US-Mexico border suggest that participants experienced multiple benefits from their research interactions with the CHW-Rs. While these findings are based on brief responses that do not reflect comprehensive study experiences, they do indicate that the relational container of the study afforded elements of a therapeutic social landscape. These gains were noted in three primary areas: experiences of care and emotional support, opportunities for novel self-reflection, and the practical acquisition of health information and individualized health results. Furthermore, the perceived impacts of these factors appeared to be amplified by participants’ preexisting barriers to healthcare as well as the backdrop of social isolation resulting from the COVID-19 pandemic.

**Figure 1:**
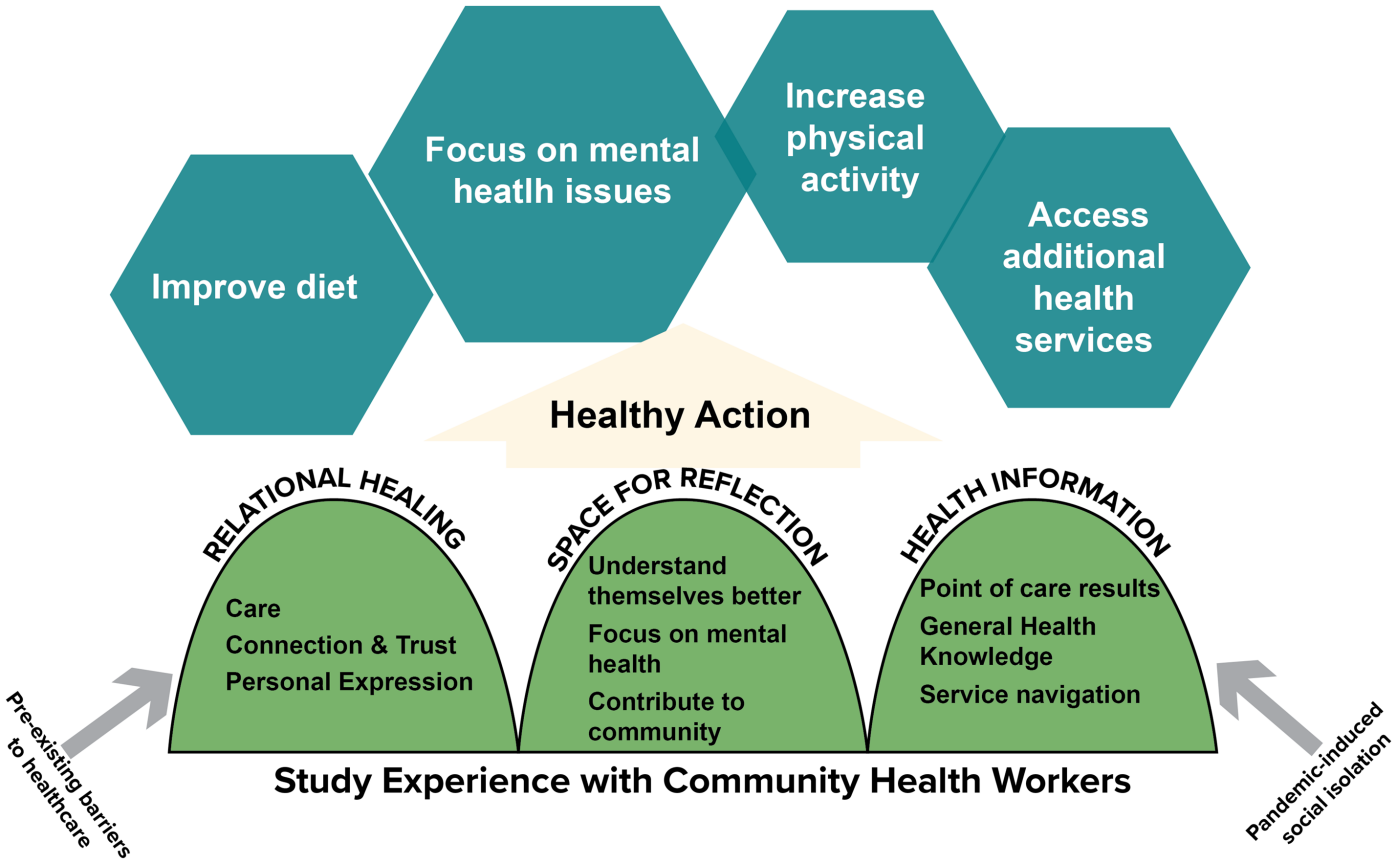
Conceptual framework of relational study experience with community health workers.

Taken in concert, participants’ positive study experiences appeared to have mobilized them to take tangible action intended to improve their own health and wellbeing, including changes to diet, increasing physical activity, accessing more medical care, and focusing more on their mental health. However, given the exploratory nature of this analysis, they cannot be understood as evidence of measurable health improvements resulting from study participation. Figure 1 provides a conceptual framework for how we leverage the therapeutic landscape theory to interpret the perceived impacts of this relationally driven CHW-led study.

The phenomena of participants perceiving significant benefit from the research experience despite the absence of planned behavior change or novel therapy interventions is an important yet understudied topic. Prior research has demonstrated that observational study components can significantly impact study participants in the areas of increasing health knowledge and investment in one’s health, particularly when participants lack regular access to medical care (49). Furthermore, intervention researchers have noted that control arm participants may experience unplanned health benefits resulting from interviewer interactions and measurements, some of which has been tied to the experience of relational support (50–52).

Here we leverage the theory of therapeutic landscape to deepen our understanding of the factors at work when non-interventional studies that incorporate CHW-Rs result in significant perceived benefits to participants. While calculating concrete changes to health outcomes may be the most seemingly objective way to “measure” study impact on participants, prior scholarship on therapeutic landscapes posits that positive perception alone, or the way one experiences a space, is a sufficient mechanism via which landscapes can help produce a healing effect (25, 53, 54). “Social landscapes” that afford opportunities for therapeutic social interaction and support can compose an integral piece of these perceived gains (55). While the therapeutic landscape theory also incorporates spatial and symbolic elements, the current study only considers the role of social and relational elements of healing since these were the topics raised by participants.

Our analysis identified an important relational component of participants’ study experience that centered around how the CHW-Rs developed an environment of care and trust with participants, producing “spaces of care” (22, 56). This multi-layered emotional connection encouraged participants to feel valued and to reflect and share openly about their lives, reducing their sense of isolation and affording them a sense of relief and lightness (34, 42).

Notably, personality attributes that increase social connection, such as being friendly, caring, and warm and having good communication skills have been found to be among the most desired skills in the CHW hiring process and “social support” is a core function of the CHW workforce (1, 57).

Participants noted that the study visits were characterized by a caring environment whether they occurred in the home or social service office setting, indicating that their personal relationships with study staff were paramount (58). In the context of healing spaces carved out in public spaces by unhoused individuals, Johnson & Light (25) argue that the conceptualization of unconventional sites as therapeutic landscapes “prompts consideration of whose values of health and healing are represented in identifying, designing, and supporting therapeutic space” (p. 122).

Our data also indicate that the background context of the COVID-19 pandemic and associated social isolation that was in effect during the data gathering period likely intensified the perceived therapeutic nature of the study’s social landscape. Social isolation and loneliness skyrocketed during the pandemic, and these conditions were further intensified along the US-Mexico border, where federally mandated closures and reduced hours at points of entry reduced opportunities for border residents to travel to connect with family, community, and cultural heritage (32, 59, 60). For immigrant populations in the US, prior scholarship has also suggested that places that offer social support and acceptance in their own language are important locales of healing and recovery (24, 26, 40). Based on a study to promote care-seeking among Latina victims of interpersonal violence, Terrazas-Carrillo et al. (61) emphasize the importance of creating psychological and physical safety within the context of shared cultural values. They argue that: “places of social support are therapeutic landscapes because healing occurs within places that hold these social networks” (61), p. 513.

Other culturally specific factors such as health definitions and concepts likewise play a critical role in determining the transformational capacity of healing spaces and community-based health approaches (62, 63). Prior research has affirmed that people of Mexican origin tend to hold integrated conceptualizations of their health in which their human relationships and the environments in which they are embedded play a determining role in their overall health (33, 64). This integrated healing worldview may be linked to why the relational aspects of this study appear to have been motivational for participants in mitigating the impacts of documented barriers to physical and mental health services among Latinos in the US (65).

The caring atmosphere established by the CHW-Rs ignited a novel process of reflection and self-discovery among participants, most of whom had never utilized mental health services. A CHW-R published testimony cited that: “During the survey, we could see on their faces where people started to reflect on the questions, like “How many close friends do you feel comfortable talking with about your private matters?” I was surprised to find that some people said they only had one or two people. I could see the sadness in their eyes” (34), p. 244. While sometimes challenging emotionally, these self-reflections were meaningful to how participants understood their own health and wellbeing.

The personal relationships established during the study also facilitated the exchange of tangible health-related tools and knowledge, which participants cited as being helpful in their process of health seeking. Prior research on the place-health link in the context of consumer experiences has demonstrated that places can serve as a “repository of resources,” where the availability of social and informational resources can have a healing effect (23, 73, p. 281). Participants cited benefitting significantly from receiving individualized results and that this information filled in significant gaps in their understanding of their own health (35).

Prior research has likewise suggested that emotions can play a critical role in determining health related and other action (66, 67). However, a persistent research gap remains in what we know about how emotional experiences in the context of research and health promotion may serve to motivate the successful application of health information to inform positive action (68).

Research on the therapeutic potential of place has argued that spaces become sites of health promotion when they contain the “networks and associations that generate the resources and agencies needed to maintain health” (55), p. 6 (69). These resources include both concrete offerings as well as social and emotional ties (58), all of which participants cited experiencing during the current study. Many of these efforts were carried out in home and informal settings, which is an arena of particular importance in Mexican immigrant populations given their historically high rates of self-care and lay medical practices (70, 71).

Our exploratory analysis of the closing interview data suggests that the study visits with CHW-Rs ignited a feedback loop in which participants felt more informed and capable to take concrete steps to improve their health. Knowing that the CHW-Rs would return to repeat the survey questions may have further reinforced participants’ motivation to make changes in the areas of increased medical care utilization and efforts to improve their dietary habits, increase physical activity levels, and prioritize their mental health. Whether participants’ positive health actions required additional support from the CHW-Rs or were actualized independently, these tangible responses to study participation reflect the development of increased health related agency among participants.

Notably, the study’s impact on participant’s perceived agency to make health improve extended beyond their own lives and also enhanced their interest in and commitment to community participation in health-related research, a stated goal of community-based research (72). Many participants stressed the importance of contributing to a study that would expand understanding of local sources of stress and therefore result in more services for the community and enact lasting change. In a study on therapeutic landscapes among the Māori of New Zealand, researchers concluded that factors of communal well-being and social justice were “critical for health, happiness and wellbeing and should not be addressed separately” (62), p. 18.

### 4.1 Strengths and limitations

To our knowledge, this exploratory analysis is the first to consider how CHW-Rs may create therapeutic landscapes for study participants, and as such offers an important contribution to our understanding of how studies may positively impact research participants. However, it has several limitations, and future research is necessary to capture comprehensive participant experiences in CHW-R studies. First, since the closing interview questions were administered by the same CHW-Rs who carried out the study, participants may have felt inclined to highlight the study’s positive impact, and in this way the uniformly positive responses may reflect reporting bias. Second, given the deductive application of the therapeutic landscape framework in a later analytic phase raises, this study primarily explores the social environment established during the study since interview questions did not produce data that would be relevant for the consideration of the impacts of physical or symbolic factors that are also cornerstones of the therapeutic landscape theory. In addition, the post-hoc nature of the theoretical construct could have introduced potential confirmation bias; however, we did ensure to incorporate all elements of the data in our analysis and allowed themes to emerge naturally during all stages of analysis. In addition, this study relies on brief responses in the context of a larger quantitative survey and thus lacks deeper exploration via tools of qualitative observation that could provide a “thick description” of participants’ therapeutic study experiences (22). Lastly, the study sample consisted of Mexican-origin adults living near the border in rural Yuma County, Arizona, and were mostly female and foreign born, limiting the generalizability of our findings to other immigrant populations and geographic contexts.

## 5 Conclusions and applied implications

This article uses the lens of therapeutic landscape to explore how CHW-Rs nurture trust and connection with research participants which provides them a fertile and safe ground for the acquisition of health tools and information as well as personal growth and change. This work contributes to the identification that CHW-Rs increase the quality of community research because they know how to develop a relationally driven study environment in which they commit to take follow-up actions and make vital links to services on issues that arise for individual community members during research. These gains in the context of both interventional and observational research carry significant potential to bolster the inclusion of immigrant and displaced populations in the research endeavor who have been under-appreciated for how they can contribute to our understanding of complex issues (18). However, these findings also raise the concern that relationally driven research led by CHW-Rs may “muddy” research results by driving unintended changes to health and wellbeing among participants in non-intervention studies or control arms and thereby add complexity to data analysis.

## Data Availability

The raw data supporting the conclusions of this article will be made available by the authors, without undue reservation.
